# Neural Correlates of Impaired Vision in Adolescents Born Extremely Preterm and/or Extremely Low Birthweight

**DOI:** 10.1371/journal.pone.0093188

**Published:** 2014-03-24

**Authors:** Claire E. Kelly, Jeanie L. Y. Cheong, Carly Molloy, Peter J. Anderson, Katherine J. Lee, Alice C. Burnett, Alan Connelly, Lex W. Doyle, Deanne K. Thompson, for the Victorian Infant Collaborative Study Group

**Affiliations:** 1 Murdoch Childrens Research Institute, Melbourne, Australia; 2 Royal Women's Hospital, Melbourne, Australia; 3 Department of Obstetrics and Gynaecology, The University of Melbourne, Melbourne, Australia; 4 Department of Paediatrics, The University of Melbourne, Melbourne, Australia; 5 School of Psychological Sciences, The University of Melbourne, Melbourne, Australia; 6 Florey Institute of Neuroscience and Mental Health, Melbourne, Australia; Vanderbilt University, United States of America

## Abstract

**Background:**

Adolescents born extremely preterm (EP; <28 weeks' gestation) and/or extremely low birthweight (ELBW; <1000 g) experience high rates of visual impairments, however the potential neural correlates of visual impairments in EP/ELBW adolescents require further investigation. This study aimed to: 1) compare optic radiation and primary visual cortical structure between EP/ELBW adolescents and normal birthweight controls; 2) investigate associations between perinatal factors and optic radiation and primary visual cortical structure in EP/ELBW adolescents; 3) investigate associations between optic radiation and primary visual cortical structure in EP/ELBW adolescents and the odds of impaired vision.

**Methods:**

196 EP/ELBW adolescents and 143 controls underwent magnetic resonance imaging at a mean age of 18 years. Optic radiations were delineated using constrained spherical deconvolution based probabilistic tractography. Primary visual cortices were segmented using FreeSurfer software. Diffusion tensor variables and tract volume of the optic radiations, as well as volume, surface area and thickness of the primary visual cortices, were estimated.

**Results:**

Axial, radial and mean diffusivities within the optic radiations, and primary visual cortical thickness, were higher in the EP/ELBW adolescents than controls. Within EP/ELBW adolescents, postnatal corticosteroid exposure was associated with altered optic radiation diffusion values and lower tract volume, while decreasing gestational age at birth was associated with increased primary visual cortical volume, area and thickness. Furthermore, decreasing optic radiation fractional anisotropy and tract volume, and increasing optic radiation diffusivity in EP/ELBW adolescents were associated with increased odds of impaired vision, whereas primary visual cortical measures were not associated with the odds of impaired vision.

**Conclusions:**

Optic radiation and primary visual cortical structure are altered in EP/ELBW adolescents compared with controls, with the greatest alterations seen in those exposed to postnatal corticosteroids and those born earliest. Structural alterations to the optic radiations may increase the risk of impaired vision in EP/ELBW adolescents.

## Introduction

Around 50% of infants born extremely preterm (EP; <28 weeks' gestation) and/or extremely low birthweight (ELBW; <1000 g) experience long-term impairment in one or more visual functions, such as visual acuity, stereopsis (binocular depth perception), vergence eye movements or visual perception [1]–[4]. Several perinatal factors have been associated with visual impairment in EP/ELBW children, including retinopathy of prematurity (ROP), major brain injury [intraventricular hemorrhage grade 3/4 or cystic periventricular leukomalacia] [5], [6], and exposure to postnatal corticosteroids, which are used to treat chronic lung disease but may have adverse side effects on neural structure and function [7]. Despite the documented associations between perinatal factors and later visual impairments in EP/ELBW children, visual impairments can arise even in the absence of a history of complications such as ROP and major brain injury [8], [9]. This suggests that additional factors may be associated with visual impairment in EP/ELBW children.

Neurohistopathological and magnetic resonance imaging (MRI) studies have highlighted the susceptibility of the preterm brain to cerebral white matter and cortical gray matter injuries [10]. Altered patterns of cortical volume, surface area and thickness have been described using structural MRI in preterm children and adolescents compared with term-born controls, and have been associated with adverse neurodevelopmental outcomes [11]–[13]. Characteristics of white matter microstructure, such as fiber density and myelination, can be studied indirectly using diffusion-weighted MRI and diffusion tensor parameters such as the fractional anisotropy (FA). Using diffusion-weighted MRI, previous studies have shown that lower FA within the major white matter tracts of the visual pathways, the optic radiations, is associated with impaired visual function in neonates born preterm [14]–[16]. However, data are limited concerning relationships between optic radiation and primary visual cortex (V1) structure in older EP/ELBW children and contemporaneous visual function.

The current study aimed to address this knowledge gap, as well as to expand upon previous studies by assessing a large and representative sample of EP/ELBW adolescents and concurrent normal birthweight (>2499 g) control adolescents, followed up from birth. This study also aimed to utilize constrained spherical deconvolution (CSD), an advanced diffusion-weighted MRI analysis method that resolves multiple white matter fiber orientations within image voxels, enabling robust delineation of white matter tracts, such as the optic radiations, even in crossing fiber regions [17]. Specific aims of the current study were to: 1) compare optic radiation and V1 structure between adolescents born EP/ELBW and normal birthweight; 2) investigate associations between perinatal variables and optic radiation and V1 structure in EP/ELBW adolescents; 3) investigate associations between optic radiation and V1 structure in EP/ELBW adolescents and the odds of impaired vision. It was hypothesized that optic radiation and V1 structure would be altered in EP/ELBW adolescents compared with controls, that perinatal complications would be associated with altered optic radiation and V1 structure in EP/ELBW adolescents, and that altered optic radiation and V1 structure in EP/ELBW adolescents would be associated with increased odds of impaired vision.

## Methods

### Participants

**Figure 1:**
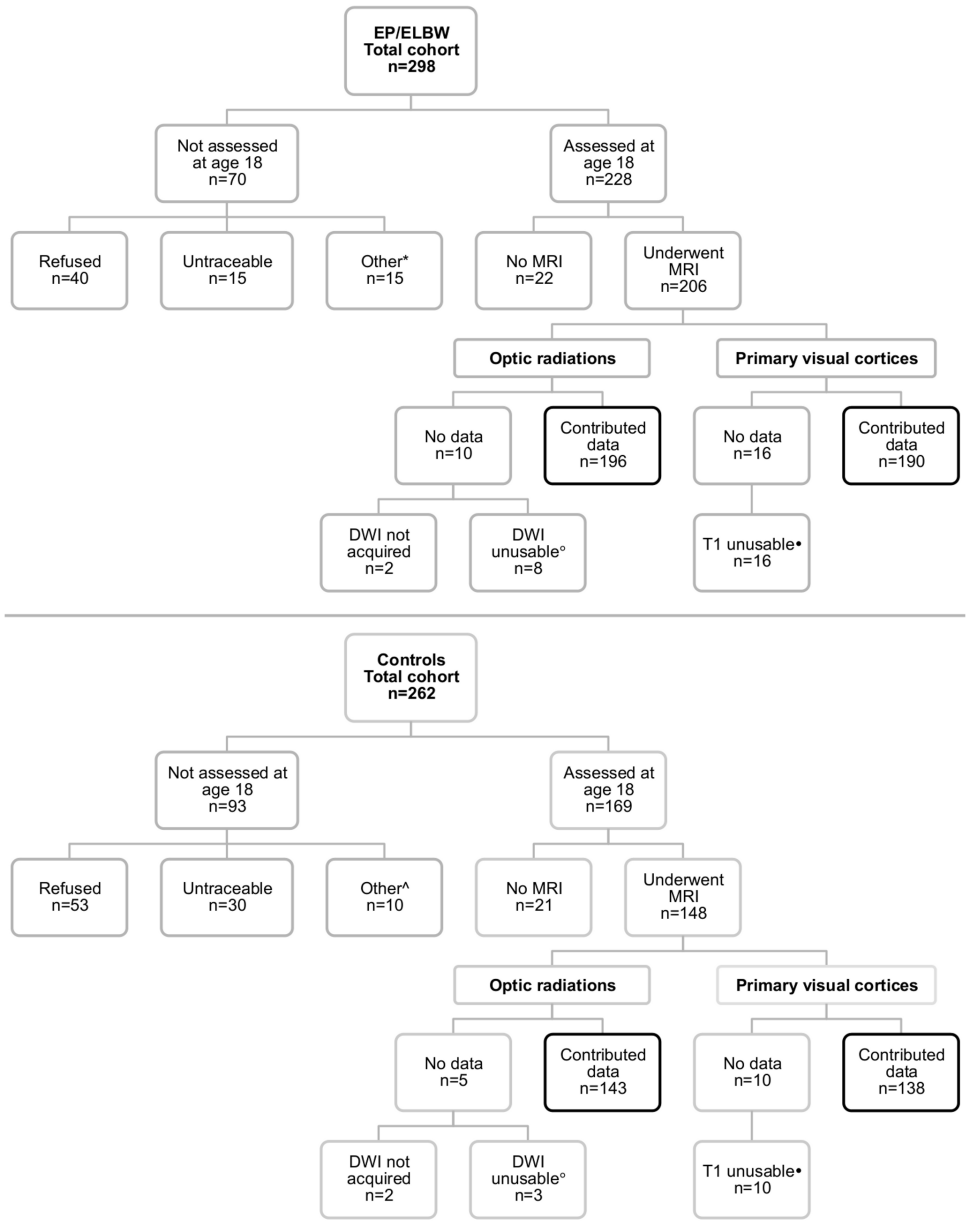
Breakdown of participant attrition and exclusion at the 18-year follow-up study. DWI =  diffusion-weighted images; ELBW =  extremely low birthweight; EP =  extremely preterm; MRI =  magnetic resonance imaging; T1 =  T1-weighted images. *Died (n = 1), too disabled to attend the assessment (n = 7), interstate/international (n = 3), unknown reason for not attending the assessment (n = 4). ∧Died (n = 2), too disabled to attend the assessment (n = 3), interstate/international (n = 5). °DWI were unusable due to movement artefact (n = 4), signal dropout due to dental braces (n = 6) or incomplete acquisitions (n = 1). ?T1-weighted images were unusable due to artefact or major cerebral structural abnormalities that affected registration during the FreeSurfer processing pipeline.

Participants were from a population-based study of all 298 survivors born EP (<28 weeks' gestation) and/or ELBW (<1000 g) between January 1991 and December 1992 in the state of Victoria, Australia. Controls were derived from a cohort of 262 infants born normal birthweight (>2499 g) and contemporaneously recruited from the three tertiary Victorian perinatal centers. Controls were matched to EP/ELBW survivors for expected date of birth, sex, mother's health insurance status and mother's country of origin. The cohorts have been assessed at 2 [18], 5 [19] and 8 [20] years of age. At approximately 18 years of age, all participants were invited to attend an extensive health and developmental assessment, including visual function assessments and MRI. Figure 1 details reasons for participant attrition and exclusions at the 18 year follow-up.

### Ethics statement

Ethical approval for the original and all follow-up studies was obtained from the Human Research and Ethics Committees of all participating hospitals (the Royal Women's Hospital, Mercy Hospital for Women, Monash Medical Centre and the Royal Children's Hospital, Melbourne). Informed written consent was obtained from all participants, as well as from parents if the participant was younger than 18 years of age at the time of assessment.

### Visual assessments

Visual assessments were performed by an examiner blind to clinical history. Participants wore habitual corrective eyewear, if required (e.g. glasses). Visual acuity was assessed monocularly using the 3 m Early Treatment Diabetic Retinopathy Study (ETDRS) logMAR chart [21]. Participants scoring ≥0.2 (Snellen equivalent 6/9 or 20/32) in the better eye were classified as impaired. Stereopsis (binocular depth perception) was assessed using the Randot Stereotest [22]; impairment was defined as a resolution ≥70 seconds of arc [23]. Vergence (simultaneous movement of both eyes in opposite directions) was assessed with the Prentice card from the Phoria test [24]; impairment was defined as a near point of convergence ≥7 cm [25]. Five aspects of visual-spatial perception were assessed with the Test of Visual-Perceptual Skills 3^rd^ Edition: visual discrimination, visual-spatial relationships, visual figure-ground, visual form constancy and visual closure [26]. Impairment in each visual perception subtest was defined as >1.5 standard deviations below the normative mean. A total visual perception score was calculated based on the scaled scores of the five subtests, and impairment was defined as <10^th^ percentile of the control group. Participants who were too visually impaired to complete a visual assessment were subsequently classified as impaired for that assessment.

### MRI acquisition

MRI was performed using a 3 T Siemens Magnetom Trio, Tim system. T1-weighted images were acquired with 3D MPRAGE sequences [voxel size = 0.7×0.7×1.2 mm^3^; coronal slices; echo time (TE)/repetition time (TR) = 2.67/1800 ms; field of view = 230×230 mm; matrix size = 320×320]. Diffusion-weighted images were acquired with echo planar imaging sequences (voxel size = 2.5 mm^3^; axial slices; TE = 110 ms; field of view = 240×240 mm; matrix size = 96×96) at two gradient strengths: 1. *b* = 1000 s/mm^2^, 25 gradient directions, TR = 8800 ms; 2. *b* = 3000 s/mm^2^, 45 gradient directions, TR = 8100 ms.

### Optic radiation tractography

**Figure 2:**
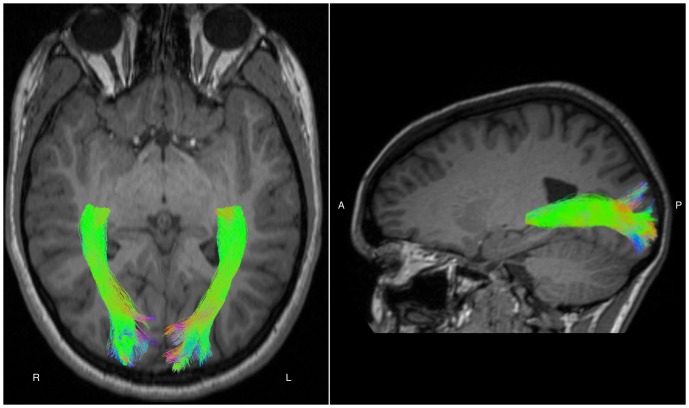
Examples of the optic radiations delineated using probabilistic tractography based on constrained spherical deconvolution. R =  right; L =  left; A =  anterior; P =  posterior.

CSD was computed on the *b* = 3000 s/mm^2^ diffusion-weighted data using MRtrix software [17], with a maximum harmonic order of 6. Using CSD's fiber orientations, optic radiations were delineated by generating streamlines probabilistically from seeds placed slightly lateral to the lateral geniculate nuclei (LGN), by an operator blind to clinical history (Figure 2). The LGN were identified on an axial slice depicting the transition from the posterior limb of the internal capsule to the cerebral peduncle, as described previously [27]. Streamlines were retained only if they entered target regions placed just anterior to V1, passed anterior-posteriorly from the seeds, entered voxels with CSD orientation amplitudes >0.2, and contained angles with <2 mm radii of curvature. 1000 streamlines were selected. Extraneous voxels containing <5/1000 streamlines were discarded.

Diffusion tensor values were obtained for within the optic radiations by multiplying the binary tract volumes by diffusion tensor maps that had been generated from the b = 1000 s/mm^2^ data and registered to the b = 3000 s/mm^2^ data using the linear image registration tool (FLIRT) within the functional MRI of the brain software library (FSL) [28]. The b = 1000 s/mm^2^ diffusion tensor maps [FA, axial diffusivity (λ_ll_), radial diffusivity (λ_⊥_) and mean diffusivity (MD)] were generated using the weighted linear least squares method within ExploreDTI, version 4.8.2. [29]. Pre-processing of the b = 1000 s/mm^2^ data within ExploreDTI involved correction for motion and eddy current induced distortions, and appropriate B-matrix re-orientation [30]. The reasons for obtaining diffusion tensor values from the co-registered b = 1000 s/mm^2^ maps rather than directly from the b = 3000 s/mm^2^ maps were: 1) Diffusion tensor values, particularly MD, vary according to the strength and number of b-values in the acquisition [31]; 2) Increasing b-values are accompanied by decreasing signal-to-noise ratios [32]. As such, the b = 1000 s/mm^2^ sequences may be expected to provide more accurate estimates of the diffusion tensor values than the b = 3000 s/mm^2^ sequences. All co-registered images were visually examined for registration errors. Diffusion tensor variables (FA, λ_ll_, λ_⊥_, MD) were obtained for the average of all voxels in each of the left and right hemisphere optic radiations. Additionally, tract volume was estimated as the number of voxels in the binary tract volumes multiplied by the voxel size. Intra-class correlations, averaged for the left and right hemisphere optic radiations, for tractography repeated on 10 cases were ≥0.99 for FA, λ_ll_, λ_⊥_ and MD and 0.89 for tract volume, indicating high intra-rater reliability.

### Primary visual cortex segmentation

Cortical reconstruction and volumetric segmentation based on the T1-weighted data was performed with the FreeSurfer image analysis suite, version 5.0 (http://surfer.nmr.mgh.harvard.edu/) [33]. The cerebral cortex was subdivided into gyral-based regions of interest [34]. During the processing pipeline, data were visually inspected and manually edited as required, by an operator blind to clinical history. Estimates of cortical volume, surface area and thickness [35] were extracted from the left and right hemisphere pericalcarine cortices, which are the anatomical locations of V1.

Intracranial volumes were also segmented using FreeSurfer, or using Statistical Parametric Mapping software if segmentation using FreeSurfer was not possible due to inadequate image quality (n = 10). Given that overall head size has been shown to influence white matter diffusion tensor values due to differential partial volume effects [36], intracranial volumes were used to control for head size related partial volume effects on diffusion tensor values.

### Statistical analysis

Data were analyzed using SPSS version 20.0. Perinatal characteristics of the participants who contributed optic radiation or V1 data and the non-participants who did not contribute data were compared using t-tests and chi-squared tests for continuous and categorical variables respectively. Baseline characteristics and visual outcomes of the EP/ELBW and control participants were also compared in a similar manner.

Optic radiation and V1 variables in the left and right hemispheres of the brain were compared between the EP/ELBW and control participants using linear regression fitted to both hemispheres simultaneously. Modelling was carried out using generalized estimating equations to account for within-subject correlations between data from the left and right hemispheres. The regressions were performed with robust (Huber/White/sandwich) estimation of covariance and with an exchangeable correlation structure. The regressions were also performed with and without including corrected age at MRI and intracranial volume in the models as covariates, to adjust for their potential effect. Interactions between birth group and hemisphere were checked for, to assess whether the results differed by hemisphere.

Amongst EP/ELBW participants, associations between perinatal variables and optic radiation and V1 variables in the left and right hemispheres of the brain were investigated using linear regression, again fitted using generalized estimating equations with robust covariance estimation and an exchangeable correlation structure, to account for the multiple observations for each individual. The perinatal variables considered were gestational age (GA) at birth, birthweight standard deviation score (BWSDS [37]), male sex, major brain injury as diagnosed by cranial ultrasound in the newborn period (intraventricular hemorrhage grade 3/4 and/or cystic periventricular leukomalacia), postnatal corticosteroid exposure and severe ROP (≥stage 3 in either eye). Firstly, each perinatal variable was entered into separate univariable regression models. Secondly, all perinatal variables were entered simultaneously into a single regression model, along with corrected age at MRI and intracranial volume, so that the effect of each perinatal variable was adjusted for the effects of all the other variables in the model, to investigate independent predictors.

Finally, associations between optic radiation and V1 variables in EP/ELBW participants and the odds of impaired visual acuity, stereopsis, vergence and visual perception were investigated using logistic regression, with separate regression models for the left and right hemisphere variables. The regressions were performed with and without including corrected age at MRI and intracranial volume in the models as covariates to adjust for their potential effect.

Given the multiple comparisons performed, the results were interpreted based on overall patterns and magnitudes of differences, rather than individual p-values.

## Results

### Participants

Optic radiation data could be analyzed for 196 EP/ELBW participants and 143 controls. Perinatal characteristics were similar between the EP/ELBW participants who contributed optic radiation data and the EP/ELBW non-participants, except fewer EP/ELBW participants had cystic periventricular leukomalacia (4% vs. 10%, p = 0.05). Baseline characteristics were similar between control participants and non-participants, except there were fewer male control participants (42% vs. 55%, p = 0.03). V1 data could be analyzed for 190 EP/ELBW participants and 138 controls. Differences between the participants who contributed V1 data and non-participants were similar to differences between participants who contributed optic radiation data and non-participants (data not shown).

Amongst participants with optic radiation data, the proportion of males was similar between the EP/ELBW and control groups, but there were expected differences in other perinatal variables (Table 1). Additionally, more EP/ELBW participants than controls had impaired visual function in most areas assessed, as expected (Table 2). Differences in perinatal characteristics and visual outcomes between the EP/ELBW participants and controls with V1 data were similar to those with optic radiation data (data not shown).

**Table 1:** Characteristics of the extremely preterm/extremely low birthweight and control participants with optic radiation data.

	EP/ELBW, n = 196	Controls, n = 143
Gestational age at birth (weeks), mean (SD)	26.6 (2.0)	39.2 (1.5)
Birthweight (g), mean (SD)	894 (161)	3423 (458)
Birthweight SD score, mean (SD)	−0.7 (1.2)	0.08 (0.9)
Male, n (%)	90 (46)	60 (42)
Postnatal corticosteroids, n (%)	60 (31)	0 (0)
Intraventricular hemorrhage grade 3/4, n (%)	12 (6)	0 (0)
Cystic periventricular leukomalacia, n (%)	8 (4)	0 (0)
Retinopathy of prematurity ≥stage 3 either eye, n (%)	22 (12)^a^	0 (0)
Corrected age at MRI (years), mean (SD)	18.2 (0.8)	18.1 (0.8)
Intracranial volume (cm^3^), mean (SD)	1439 (160)^b^	1524 (168)^c^

ELBW =  extremely low birth weight; EP =  extremely preterm; MRI =  magnetic resonance imaging; SD =  standard deviation. ^a^n = 191; ^b^n = 194; ^c^n = 140.

**Table 2:** Visual outcomes of the extremely preterm/extremely low birthweight and control participants with optic radiation data.

Visual test	Impaired, n (%)		Odds ratio (95% CI)	p
	EP/ELBW	Controls		
Visual acuity (log score)	37 (19)^a^	26 (18)^b^	0.9 (0.5, 1.7)	0.8
Stereopsis (seconds of arc)	43 (23)^c^	12 (9)^b^	0.3 (0.2, 0.6)	0.001
Vergence	32 (16)^d^	10 (7)^e^	0.4 (0.2, 0.8)	0.01
Total visual perception score	42 (22)^d^	12 (9)^b^	0.3 (0.2, 0.7)	0.001

ELBW =  extremely low birth weight; EP =  extremely preterm; SD =  standard deviation.

a n = 192; ^b^n = 141; ^c^n = 191; ^d^n = 194; ^e^n = 138.

### EP/ELBW participants vs. controls: optic radiation and V1 measures

Optic radiation volume and FA were similar between the EP/ELBW and control participants. However, optic radiation λ_ll_, λ_⊥_ and MD were higher in the EP/ELBW participants than controls, even after adjustment for corrected age at MRI and intracranial volume (Table 3). There was little evidence of interactions between birth group and hemisphere for the optic radiation variables (all p-values>0.05).

**Table 3:** Optic radiation and primary visual cortical data in the extremely preterm/extremely low birthweight participants vs. controls.

MRI variable	EP/ELBW		Controls		Mean difference*	95% CI		p
	Mean	SD	Mean	SD		Lower	Upper	
OR FA	0.45	0.02	0.45	0.02	0.0002	−0.004	0.005	0.9
OR λ_ll_ (×10^−3^ mm^2^/s)	1.37	0.09	1.32	0.05	0.058	0.043	0.072	<0.001
OR λ_⊥_ (×10^−3^ mm^2^/s)	0.67	0.07	0.63	0.04	0.044	0.033	0.055	<0.001
OR MD (×10^−3^ mm^2^/s)	0.91	0.07	0.86	0.04	0.048	0.037	0.06	<0.001
OR volume (cm^3^)	13.07	1.64	13.14	1.5	−0.029	−0.362	0.305	0.9
V1 volume (cm^3^)	2.19	0.41	2.24	0.43	0.057	−0.027	0.142	0.2
V1 area (cm^2^)	13.92	2.23	14.8	2.65	−0.252	−0.742	0.237	0.3
V1 thickness (mm)	1.71	0.12	1.66	0.1	0.055	0.030	0.080	<0.001

CI =  confidence interval; ELBW =  extremely low birthweight; EP =  extremely preterm; FA =  fractional anisotropy; MD =  mean diffusivity; MRI =  magnetic resonance imaging; OR =  optic radiation; SD =  standard deviation; V1 =  primary visual cortex; λ_ll_  =  axial diffusivity; λ_⊥_ =  radial diffusivity. *Adjusted for corrected age at MRI and intracranial volume.

V1 volume was similar between the groups (Table 3). V1 area was lower in the EP/ELBW participants than controls in the unadjusted analysis [mean difference (95% confidence interval (CI)): −0.9 (−1.4, −0.3) cm^2^, p = 0.002], however this difference was less marked and the evidence was weaker after adjusting for corrected age at MRI and intracranial volume (Table 3). V1 thickness was higher in the EP/ELBW participants than controls, even after adjustment for corrected age at MRI and intracranial volume (Table 3). There was little evidence of interactions between birth group and hemisphere for the V1 variables (all p-values>0.05).

### Associations with perinatal variables

**Figure 3:**
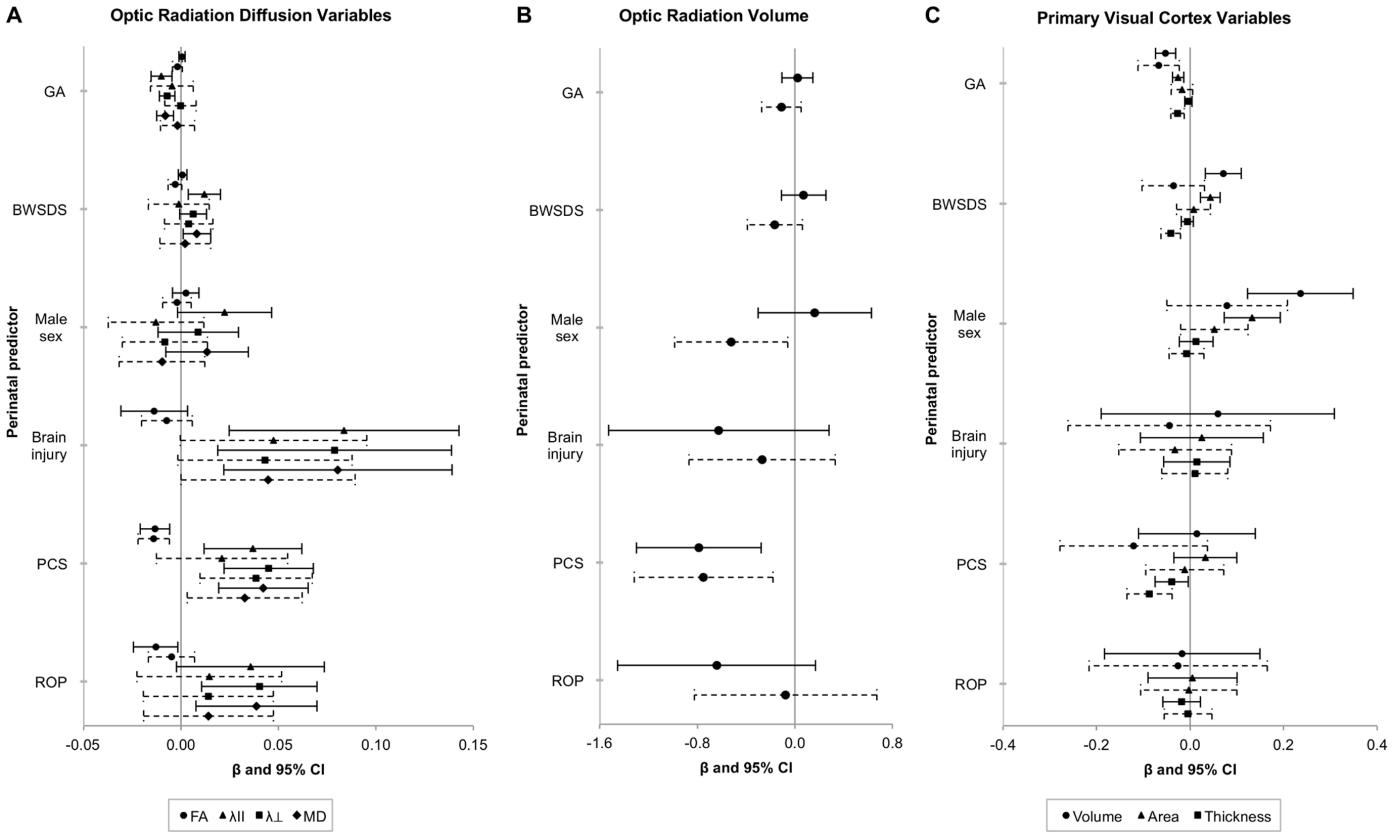
Perinatal predictors of optic radiation and primary visual cortex measures in EP/ELBW adolescents. The x-axes represent the regression coefficients and 95% confidence intervals (CI) for the associations between perinatal variables and optic radiation diffusion tensor variables (A), optic radiation volume (B), and primary visual cortical volume, area and thickness (C) in EP/ELBW adolescents, from linear regression models incorporating both the left and right hemisphere measurements. Solid lines represent the unadjusted analyses and dashed lines represent the analyses adjusted for corrected age at MRI, intracranial volume and all of the other perinatal variables. BWSDS =  birthweight standard deviation score; CI =  confidence interval; FA =  fractional anisotropy; GA =  gestational age at birth (weeks); MD =  mean diffusivity; PCS =  postnatal corticosteroid exposure; ROP =  severe retinopathy of prematurity; β =  regression coefficient (the change in the optic radiation or primary visual cortex variable per unit change in the predictor); λll =  axial diffusivity; λ⊥ =  radial diffusivity. The units of the optic radiation and primary visual cortex variables are as follows: Axial, radial and mean diffusivities, ×10^−3^ mm^2^/s; Optic radiation volume, cm^3^; Primary visual cortical volume, cm^3^; Primary visual cortical area, ×10^−1^ cm^2^; Primary visual cortical thickness, mm.

Postnatal corticosteroid exposure was associated with lower optic radiation FA and volume, and higher optic radiation diffusivity in EP/ELBW participants, even after adjustment for corrected age at MRI, intracranial volume and all of the tested perinatal variables (Figures 3A and B). There was also some evidence that decreasing GA at birth, major neonatal brain injury and severe ROP were associated with higher optic radiation λ_ll_, λ_⊥_ and MD in EP/ELBW participants, however these associations weakened in the adjusted analyses (Figure 3A). There was little evidence that BWSDS or sex were associated with the optic radiation variables in EP/ELBW participants (Figures 3A and B).

Decreasing GA at birth was associated with increasing V1 volume in EP/ELBW participants, as well as with increasing V1 area and thickness with slightly weaker evidence, even after adjustment for corrected age at MRI, intracranial volume and all of the tested perinatal variables (Figure 3C). Increasing BWSDS and male sex were associated with higher V1 volume and area in EP/ELBW participants, however these associations weakened in the adjusted analyses (Figure 3C). There was weak evidence that major neonatal brain injury, postnatal corticosteroid exposure and severe ROP were associated with V1 volume, area or thickness in EP/ELBW participants (Figure 3C).

### Associations with visual outcomes

**Figure 4:**
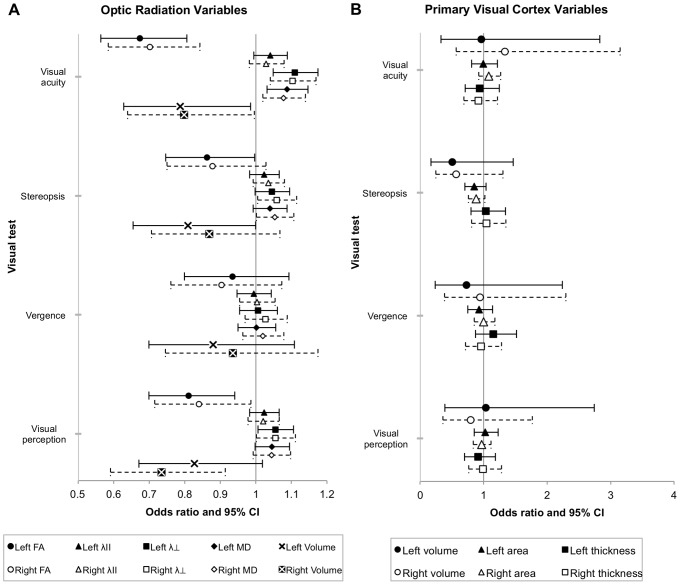
Associations between optic radiation and primary visual cortex measures in EP/ELBW adolescents and the odds of impaired visual outcomes. The x-axes represent the odds ratios and 95% confidence intervals (CI) for the associations between optic radiation variables (A) and primary visual cortex variables (B) in the left and right hemispheres of EP/ELBW adolescents and the odds of impaired visual acuity, stereopsis, vergence or visual perception, from separate logistic regression models. Solid lines represent analyses with variables from the left hemisphere of the brain; dashed lines represent analyses with variables from the right hemisphere. All results presented are adjusted for corrected age at MRI and intracranial volume. CI =  confidence interval; FA =  fractional anisotropy; MD =  mean diffusivity; λll =  axial diffusivity; λ⊥ =  radial diffusivity. The units of the optic radiation and primary visual cortex variables are as follows: Fractional anisotropy, ×100; Axial, radial and mean diffusivities, ×10^−1^ mm^2^/s; Optic radiation volume, cm^3^; Primary visual cortical volume, cm^3^; Primary visual cortical area, cm^2^; Primary visual cortical thickness, ×10 mm.

In both the left and right hemispheres of the EP/ELBW participants, increasing FA and volume and decreasing diffusivity in the optic radiations were associated with reduced odds of impaired visual acuity; adjustment for corrected age at MRI and intracranial volume had little effect (Figure 4A). There were similar patterns of associations between optic radiation variables in EP/ELBW participants and the odds of impaired stereopsis and visual perception, but with slightly smaller magnitudes and slightly weaker evidence, with even weaker evidence for vergence (Figure 4A).

There was little evidence that V1 volume, area or thickness in either the left or right hemispheres were associated with the odds of impaired visual acuity, stereopsis, vergence or visual perception in EP/ELBW participants, before or after adjusting for corrected age at MRI and intracranial volume (Figure 4B).

## Discussion

In summary, optic radiation λ_ll,_ λ_⊥_ and MD, and V1 thickness, were higher in the EP/ELBW adolescents than controls. Within the EP/ELBW adolescents, postnatal corticosteroid exposure was associated with altered optic radiation diffusion values and reduced optic radiation volume, while decreasing GA at birth was associated with increased V1 volume, and increased V1 area and thickness to a lesser extent. Finally, decreasing optic radiation FA and volume, and increasing optic radiation diffusivity within EP/ELBW adolescents were associated with increased odds of impaired visual acuity, and to a lesser extent increased odds of impaired stereopsis and visual perception.

The finding of higher λ_ll,_ λ_⊥_ and MD within the optic radiations of EP/ELBW adolescents than controls suggests that optic radiation microstructure is altered in EP/ELBW adolescents compared with controls. Higher λ_ll_, λ_⊥,_ and MD in EP/ELBW adolescents than controls may reflect altered optic radiation development, given that λ_ll_, λ_⊥,_ and MD decrease in the white matter over time in typically developing children and adolescents [38]. The λ_ll,_ λ_⊥_ and MD are thought to be sensitive to changes in one or more properties of white matter microstructure, including white matter fiber density, fiber diameter, myelination or membrane permeability [32].

Additionally, higher V1 thickness in the EP/ELBW adolescents than controls suggests that V1 structure is altered compared with controls. Cortical thickness peaks during early to middle childhood and declines thereafter in typically developing children [42]; thus the current finding may suggest that EP/ELBW birth is associated with delayed development or delayed synaptic pruning of V1. Additionally, it is possible that altered V1 structure in EP/ELBW adolescents compared with controls may to some extent reflect cortical reorganisation as a mechanism to compensate for visual impairment and early brain injury associated with EP/ELBW birth [43]. The current finding is in agreement with previous reports of increased cortical thickness in various neuroanatomical regions of children born preterm relative to controls [11], [39]–[41].

Postnatal corticosteroid exposure, major neonatal brain injury, severe ROP and decreasing GA at birth were associated with altered optic radiation diffusion values within EP/ELBW adolescents, when investigated individually. However, these associations weakened after adjusting for the remaining tested perinatal variables, corrected age at MRI and intracranial volume, with the exception of postnatal corticosteroid exposure. This finding highlights the importance of postnatal corticosteroid exposure as a predictor of altered optic radiation structure in EP/ELBW adolescents, independent of other clinically important perinatal variables. Additionally, this finding may provide an explanation for previous reports of associations between postnatal corticosteroid exposure and later visual impairments in preterm children [7]. Comparable with the current finding, previous studies have reported reduced cerebral tissue volume in preterm infants [44], [45] and in EP/ELBW adolescents [46] who were exposed to postnatal corticosteroids compared with unexposed preterm infants or adolescents, indicating a relationship between postnatal corticosteroid exposure and altered brain structure in preterm children. However, no previous studies have investigated the relationship between postnatal corticosteroid exposure and optic radiation diffusion values in preterm children.

Additionally, there was evidence that decreasing GA at birth was associated with increased V1 volume, area and thickness in EP/ELBW adolescents, independent of the other tested perinatal variables. This finding suggests that EP/ELBW adolescents who are born earliest are at greatest risk of altered V1 structure in adolescence, and may not demonstrate the normal developmental decline in synaptic density that occurs after middle childhood in typically developing children [42]. The current finding is similar to Nagy et al.'s finding of several regions of thicker cortex in adolescents born ≤28 weeks' GA compared with adolescents born >28 but ≤36 weeks' GA [11].

Importantly, altered optic radiation diffusion values and decreased optic radiation volume in EP/ELBW adolescents were associated with increased odds of impaired vision, particularly impaired visual acuity, and also impaired stereopsis and visual perception to a lesser extent. This finding suggests that optic radiation structure-function relationships with visual impairment exist in EP/ELBW adolescents. This concurs with previous reports of associations between FA within the optic radiations and visual function in preterm *infants* at term equivalent age [47] and up to 6–20 months of age [16], but extends previous studies by suggesting that optic radiation structure-function relationships with visual impairment persist in adolescence in individuals born preterm.

However, there was little evidence that optic radiation diffusion values or volume in EP/ELBW adolescents were associated with the odds of impaired vergence. Vergence, which rotates both eyes in opposite directions, may be associated with the functioning of the extra-ocular muscles or subcortical projections to brainstem neurons, rather than connectivity to or processing by the visual cortices [48]. Previously, cystic periventricular leukomalacia affecting the optic radiations has been associated with impaired eye movements [49], however the current results suggest that subtler microstructural optic radiation alterations may not preclude vergence in EP/ELBW adolescents.

Additionally, despite finding relationships between optic radiation variables and the odds of impaired vision, there was little evidence that V1 volume, area or thickness were associated with the odds of impaired vision in EP/ELBW adolescents. This suggests that V1 structure may not be sensitive to detecting structure-function relationships with impaired vision in EP/ELBW adolescents. A previous study reported associations between reduced occipital volume, encompassing gray matter from both V1 and extrastriate visual cortices, in very preterm infants at term equivalent age and impaired oculomotor function in early childhood [50]. However, no studies have investigated the relationship between V1 structure and visual function in EP/ELBW adolescents.

The current study is the first reported long-term study of optic radiation and V1 structure in EP/ELBW adolescents in relation to visual outcome. Other strengths of the current study include the large and representative sample of EP/ELBW adolescents and concurrent controls, followed up from birth. Additionally, advanced tractography based on CSD was employed, providing robust tractography results. A range of visual tests was utilized, enabling a comprehensive investigation of the potential role of the optic radiations and V1 in the visual functioning of EP/ELBW adolescents.

This study had certain limitations. Firstly, previous studies have shown that the exact size, shape and location of V1 are variable between individuals [51], and it is acknowledged that this variability may increase the likelihood of segmentation errors during the automated FreeSurfer pipeline. Additionally, while the current study focussed on V1 and its relationship to visual impairment in EP/ELBW adolescents, future studies would benefit from investigating other visual cortical regions, such as the extrastriate cortices or the LGN, to identify other potential structural abnormalities relative to controls and other potential structure-function relationships. Secondly, although the tractography results followed the known anatomical course of the optic radiations, Meyer's loop was not delineated, possibly due to limitations with the chosen seed placement [52]. Thirdly, the diffusion tensor model cannot sensitively distinguish between different aspects of white matter microstructure such as fiber density and myelination, and may also be affected by partial volume effects from non-white matter tissue and crossing fibers [32].

In conclusion, optic radiation and V1 structure are altered in EP/ELBW adolescents compared with controls. Postnatal corticosteroid exposure and decreasing GA at birth are associated with altered optic radiation and V1 structure in EP/ELBW adolescents. Furthermore, altered optic radiation structure in EP/ELBW adolescents is associated with increased odds of impaired vision, and thus may further explain the high rates of impaired vision in EP/ELBW adolescents. Conversely, V1 structure is not as sensitive to detecting structure-function relationships with impaired vision in EP/ELBW adolescents. Elucidating the neural correlates of impaired vision in EP/ELBW adolescents is an important requisite for improving visual outcome in this population.
